# A protocol for classifying normal- and flat-arched foot posture for research studies using clinical and radiographic measurements

**DOI:** 10.1186/1757-1146-2-22

**Published:** 2009-07-04

**Authors:** George S Murley, Hylton B Menz, Karl B Landorf

**Affiliations:** 1 Department of Podiatry, Faculty of Health Sciences, La Trobe University, Bundoora, Australia; 2 Musculoskeletal Research Centre, Faculty of Health Sciences, La Trobe University, Bundoora, Australia

## Abstract

**Background:**

There are several clinical and radiological methods available to classify foot posture in research, however there is no clear strategy for selecting the most appropriate measurements. Therefore, the aim of this study was to develop a foot screening protocol to distinguish between participants with normal- and flat-arched feet who would then subsequently be recruited into a series of laboratory-based gait studies.

**Methods:**

The foot posture of ninety-one asymptomatic young adults was assessed using two clinical measurements (normalised navicular height and arch index) and four radiological measurements taken from antero-posterior and lateral x-rays (talus-second metatarsal angle, talo-navicular coverage angle, calcaneal inclination angle and calcaneal-first metatarsal angle). Normative foot posture values were taken from the literature and used to recruit participants with normal-arched feet. Data from these participants were subsequently used to define the boundary between normal- and flat-arched feet. This information was then used to recruit participants with flat-arched feet. The relationship between the clinical and radiographic measures of foot posture was also explored.

**Results:**

Thirty-two participants were recruited to the normal-arched study, 31 qualified for the flat-arched study and 28 participants were classified as having neither normal- or flat-arched feet and were not suitable for either study. The values obtained from the two clinical and four radiological measurements established two clearly defined foot posture groups. Correlations among clinical and radiological measures were significant (*p *< 0.05) and ranged from *r *= 0.24 to 0.70. Interestingly, the clinical measures were more strongly associated with the radiographic angles obtained from the lateral view.

**Conclusion:**

This foot screening protocol provides a coherent strategy for researchers planning to recruit participants with normal- and flat-arched feet. However, further research is required to determine whether foot posture variations in the sagittal, transverse or both planes provide the best descriptor of the flat foot.

## Background

Foot posture, like most human anthropometric characteristics, varies considerably among children, adults and the older population [1]. Some variations in foot posture are associated with changes in lower limb motion [2,3] and muscle activity [4], and are strongly influenced by some systemic conditions, such as neurological [5] and rheumatological diseases [6]. These factors add weight to the view that functional differences exist between different foot types. Therefore, there is a need for strategies to accurately classify foot posture and define normal and potentially 'abnormal' foot types.

To address this issue, normative data are now available that classify foot posture using the following techniques: visual observation [1]; measurement of navicular height [7] or midfoot height [8]; footprint measures [7,9]; and angular measures derived from radiographs [10]. As interpretation of the clinical techniques is confounded by soft tissue overlying the skeletal structure of the foot, radiographic techniques are regarded as the gold-standard for assessing skeletal alignment of the foot in a static weightbearing position [11]. Therefore, angular foot measurements derived from x-rays are often used to validate clinical measures of foot posture [8,12,13]. As such, it would be useful to have clinical measurements that accurately predict angular measurements derived from radiographs, as this process would reduce: (i) the expense of obtaining x-rays for a study; and (ii) unnecessary referral of participants for x-ray examination.

There have already been some attempts to address this issue. Menz and Munteanu [12] evaluated the association between three clinical measurements (arch index [9], foot posture index [2], and navicular height [14]) with three lateral-view x-ray measurements (navicular height, calcaneal inclination angle, and the calcaneal-first metatarsal angle) in 95 older participants. All three clinical measures demonstrated significant correlations with the x-ray measures, with the navicular height and arch index clinical measurements having the strongest correlations. In addition, Saltzman et al. [14] investigated the association between various measures of arch height and radiological measures for 100 patients with orthopaedic conditions (mean age, 46 years). The arch height measures were all reported to have good to strong correlations with angles derived from lateral x-ray views. Other clinical measures, such as the arch ratio have also been validated using x-rays [8]. However, further research is still required to validate clinical measures with additional angles of the foot, particularly angles assessed from the anterior-posterior view, and to validate measurements specific to the young adult population.

The major drawback for researchers is that the available literature does not provide a pathway for choosing a series of clinical and radiological measurements to screen participants' foot posture. A combination of validated clinical measurements and normative data would allow researchers to have a clear protocol to follow when screening participants' foot posture, whether for laboratory-based research or epidemiological studies.

Accordingly, the primary aim of this study was to develop a foot screening protocol using clinical and radiographic measurements for the purpose of recruiting participants with normal- and flat-arched feet for a series of laboratory-based gait studies. The secondary aim was to explore relationships between the clinical and radiographic measures of foot posture.

## Methods

### Participants

Ninety-one asymptomatic young adults were recruited (45 male and 46 female) aged 18 to 47 years (mean ± SD, 23.2 ± 5.6 years) (Table 1). The participants were without symptoms of macrovascular (e.g. angina, stroke, peripheral vascular disease) and/or neuromuscular disease, or any biomechanical abnormalities which affected their ability to walk. Ethical approval was obtained for the study from the La Trobe University Human Ethics Committee (Ethics ID: FHEC06/205) and it was registered with the Radiation Safety Committee of the Victorian Department of Human Services. The x-rays were performed in accordance with the Australian Radiation Protection and Nuclear Safety Agency Code of Practice for the *Exposure of Humans to Ionizing Radiation for Research Purposes *(2005) [15].

**Table 1:** Participant anthropometric and foot posture characteristics

	**Foot posture groups**		
	
	Flat-arch n = 31	Normal-arch n = 32	Others n = 28

**General anthropometric**			
Gender ratio (female/male)	16/15	16/12	17/15
Age mean ± SD (years)	22.0 ± 4.3	23.5 ± 5.7	24.2 ± 6.7
Height mean ± SD (cm)	171.0 ± 10.0	169.7 ± 9.7	n/a
Weight mean ± SD (Kg)	73.3 ± 15.50	69.9 ± 13.6	n/a
Left or right foot count	16 right 15 left	14 right 18 left	13 right 15 left
			
**Clinical measurements**			
AI mean ± SD	0.30 ± 0.07*	0.24 ± 0.04*	0.23 ± 0.02
NNHt mean ± SD	0.18 ± 0.04^†^	0.27 ± 0.03^†^	0.25 ± 0.06
			
**Radiographic measurements**			
CIA mean ± SD (degrees)	16.1 ± 5.0^#^	20.9 ± 3.4^#^	24.9 ± 4.9
C1MA mean ± SD (degrees)	141.7 ± 6.7^‡^	132.8 ± 4.0^‡^	129.0 ± 7.7
TNCA mean ± SD (degrees)	27.5 ± 8.9^	12.5 ± 8.6^	13.0 ± 6.5
T2MA mean ± SD (degrees)	27.5 ± 10.2^¥^	13.3 ± 6.3^¥^	13.8 ± 5.3

AI – arch index, NNHt – normalised navicular height truncated, CIA – calcaneal inclination angle, C1MA – calcaneal first metatarsal angle, TNCA – talo-navicular coverage angle, T2MA – talus-second metatarsal angle.

Mean differences and 95% confidence interval (CI) expressed relative to normal-arch.

Statistically significant findings for comparisons listed below (*p *< 0.001):

* AI: mean difference 0.05, 95% CI 0.03 to 0.08

^† ^NNHt: mean difference -0.09, 95% CI -0.11 to -0.07

^# ^CIA: mean difference -4.8°, 95% CI -6.9° to -2.6°

^‡ ^C1MA: mean difference 9.0°, 95% CI 6.2° to 11.7°

^^ ^TNCA: mean difference 15.0°, 95% CI 10.7° to 19.3°

^¥ ^T2MA: mean difference 14.2°, 95% CI 9.9° to 18.4°

**Figure 1:**
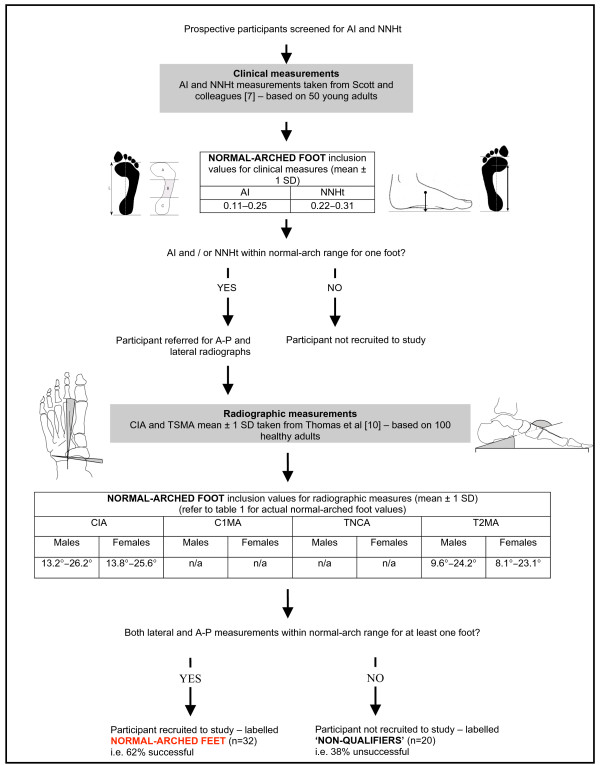
**Screening protocol for normal-arched foot posture**. Flow chart shows how the foot posture screening protocol was derived from normative data. * Values derived from Scott and colleagues [7]. CIA – calcaneal inclination angle, C1MA – calcaneal-first metatarsal angle, TNCA – talo-navicular coverage angle, T2MA – talus-second metatarsal angle.

**Figure 2:**
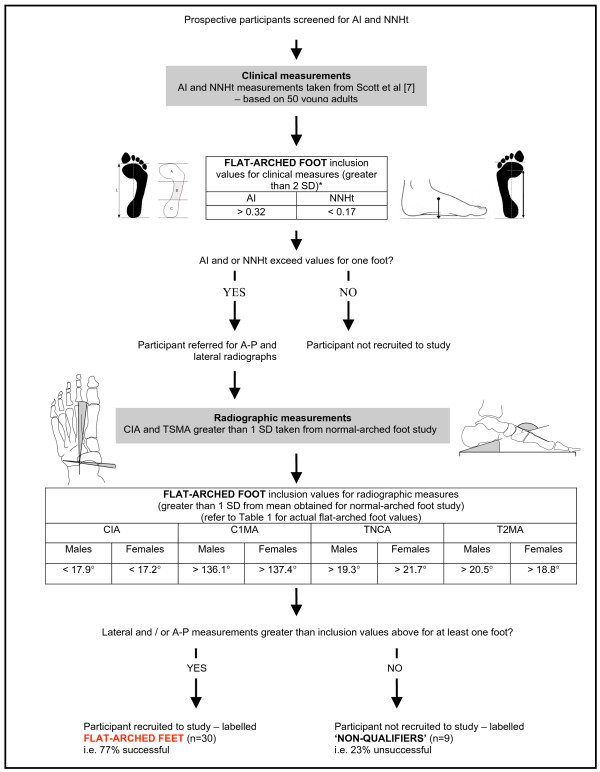
**Screening protocol for flat-arched foot posture**. Flow chart shows how the foot posture screening protocol was derived from normative data. * Values derived from Scott and colleagues [7]. The rationale for using 2 SD standard deviations was to increase the likelihood of participants with flat-arched feet qualifying for inclusion via radiographic appraisal. CIA – calcaneal inclination angle, C1MA – calcaneal-first metatarsal angle, TNCA – talo-navicular coverage angle, T2MA – talus-second metatarsal angle.

Participants were primarily recruited from the student and staff community at La Trobe University. The foot screening protocol was developed to recruit participants with normal-arched feet, which provided normative reference values for two radiographic measures of foot posture (talo-navicular coverage angle and calcaneal-first metatarsal angle). Data from these participants were subsequently used to define the boundary between normal- and flat-arched feet. This information was then used to recruit participants with flat-arched feet. Therefore, the foot screening protocol was developed by utilising: (i) published normative data for clinical and radiological measurements; and (ii) radiological measurements obtained from the first study investigating normal-arched feet (Figure 1 and 2). Participants with high-arched feet were not required for this study. Although high-arched feet are susceptive to injury and warrant greater research [16,17], this foot type is far less common than normal- and flat-arched feet [1], thus we chose to focus on two participant groups that would have greater generalisability to the wider population.

### Stage 1: Clinical measurements

The first stage of the screening protocol involved two clinical measures of foot posture; (i) the arch index [9], and (ii) normalised navicular height truncated [18]. These 'ratio' measurements have moderate to high correlations with angular measurements derived from radiographs [11,14,19], which provide the most valid representation of skeletal foot alignment [12]. Although the arch index and normalised navicular height measurements have comparable reliability to other measures of arch height, these were selected because of their ease of use and demonstrated validity with skeletal alignment measured via radiographs [12]. Additionally, the arch index is sensitive to age-related changes in foot posture [7] and is strongly associated with both maximum force and peak pressure in the midfoot during walking [20]. The primary purpose of using the clinical tests in this study was to avoid unnecessary referral of participants for radiographic assessment.

**Figure 3:**
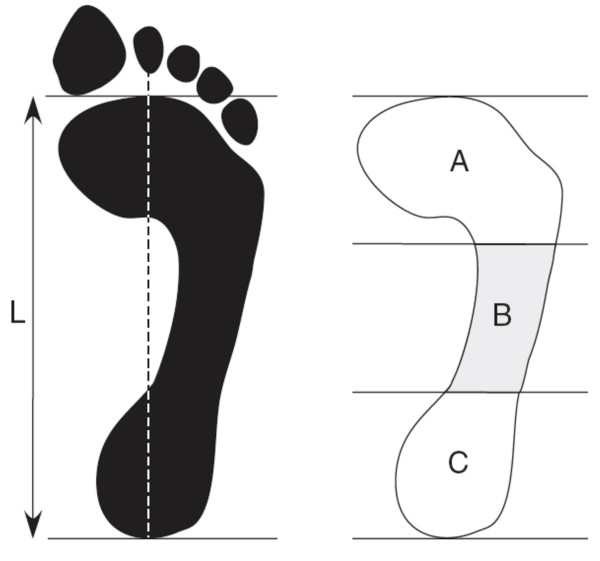
**Arch index**. Footprint with reference lines for calculating the arch index. The length of the foot (excluding the toes) is divided into equal thirds to give three regions: A – forefoot; B – midfoot; and C – heel. The arch index is then calculated by dividing the midfoot region (B) by the entire footprint area (i.e. Arch index = B/[A+B+C]).

The arch index was calculated as the ratio of area of the middle third of the footprint to the entire footprint area not including the toes, with a higher ratio indicating a flatter foot [9] (Figure 3). The footprint was taken using carbon paper and a graphics tablet was used to calculate the surface area in each third of the foot.

**Figure 4:**
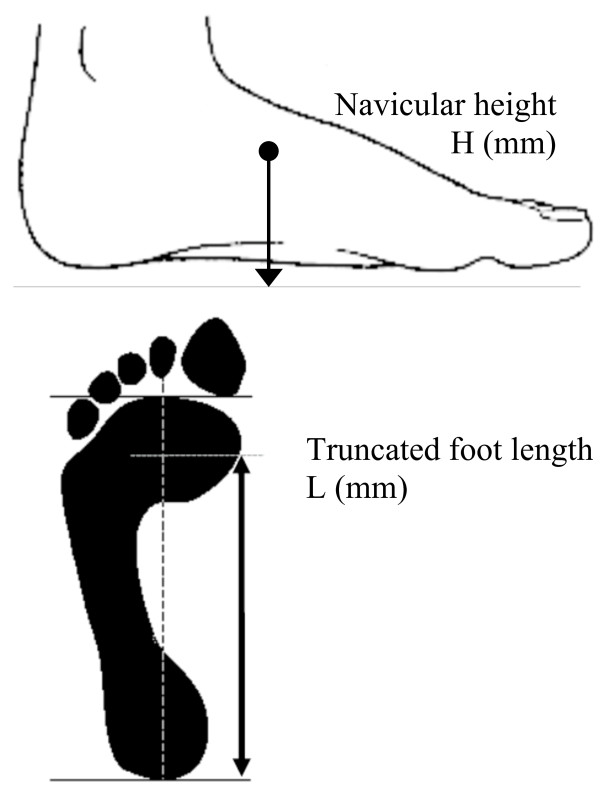
**Normalised navicular height (truncated)**. Calculating normalised navicular height truncated. The distance between the supporting surface and the navicular tuberosity is measured. Foot length is truncated by measuring the perpendicular distance from the 1^st ^metatarsophalangeal joint to the most posterior aspect of the heel. Normalised navicular height truncated is calculated by dividing the height of the navicular tuberosity from the ground (H) by the truncated foot length (L) (i.e. Normalised navicular height truncated = H/L).

Normalised navicular height truncated is the ratio of navicular height relative to the truncated length of the foot. Navicular height is the distance measured from the most medial prominence of the navicular tuberosity to the supporting surface. Foot length is truncated by measuring the perpendicular distance from the first metatarsophalangeal joint to the most posterior aspect of the heel [18], with a lower normalised navicular height ratio indicating a flatter foot (Figure 4).

To determine normal values for the arch index and normalised navicular height, we requested and were provided with raw foot posture measurements from Scott and colleagues [7] comprising data from 50 healthy young adults (26 female and 24 male with a mean age ± SD of 20.9 ± 2.6 years). The participants reported on by Scott and colleagues [7] were of similar age to the target participants for our study (Figure 1).

For the normal-arched foot study, participants qualified for the second stage of the screening assessment involving radiographic evaluation when either the arch index and normalised navicular height scores fell within ± 1 standard deviation (SD) of the mean values adapted from Scott and colleagues [7] (Figure 1). A threshold of ± 1 SD was selected as the 'normal limits' of several human physiological and anthropometric characteristics are frequently defined to lie within 1–2 standard deviations of the population mean [21].

### Stage 2: Radiographic measurements

**Figure 5:**
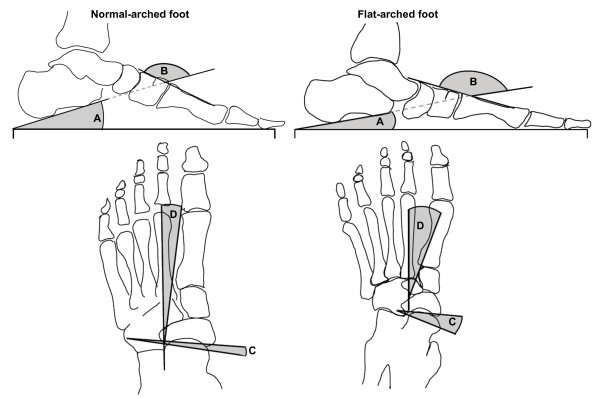
**Radiographic measurements**. Traces from two representative participants illustrate x-ray angular measurements from normal (left) and flat-arched (right) foot posture. Lateral views (top) show: calcaneal inclination angle; calcaneal-first metatarsal angle; anterior posterior views (bottom) show: talonavicular coverage angle; talus second metatarsal angle. A – calcaneal inclination angle, B – calcaneal-first metatarsal angle, C – talo-navicular coverage angle, D – talus-second metatarsal angle. Angle A *decreases *with flat-arched foot posture; angle B, C and D *increase *with flat-arched foot posture, compared to the normal-arched foot posture.

The second screening stage involved two bilateral radiographs comprising: (i) antero-posterior (A-P) and (ii) lateral views obtained with the subject weight-bearing in a relaxed bipedal stance position. From the A-P view, the talus-second metatarsal angle and the talo-navicular coverage angle were assessed (Figure 5). From the lateral view, the calcaneal inclination angle and the calcaneal-first metatarsal angle were assessed (Figure 5). These angles were chosen to represent foot posture based on: (i) ease of measurement and good reliability; and (ii) degree by which they reflect foot posture in both the sagittal and transverse planes.

#### Anterior-posterior radiographic angles

The talo-navicular coverage angle is formed by the bisection of the anterior-medial and the anterior-lateral extremes of the talar head and the bisection of the proximal articular surface of the navicular [22] (Figure 5). The talus-second metatarsal angle is formed by the bisection of the second metatarsal and a line perpendicular to a line connecting the anterior-medial and the anterior-lateral extremes of the talar head [10] (Figure 5). Angles measured from the A-P view reflect transverse plane alignment of the midfoot and forefoot, with larger angles for the talo-navicular coverage angle and talus-second metatarsal angles indicating a flatter foot.

#### Lateral radiographic angles

The calcaneal inclination angle is the angle between the inferior surface of the calcaneus and the supporting surface [14] (Figure 5). The calcaneal-first metatarsal angle is the angle formed by the inferior surface of the calcaneus and a line parallel to the dorsum of the mid-shaft of the first metatarsal. Angles measured from the lateral view reflect sagittal plane alignment of the hindfoot and forefoot, with a lower calcaneal inclination angle and a greater calcaneal-first metatarsal angle indicating a flatter foot (Figure 5).

Normal values for the calcaneal-inclination angle were derived from a study by Thomas and colleagues [10] comprising 100 adults (50 females and 50 males with a mean age of 34.7 years for females and 34.3 years for males), which represents a slightly older population to that included in our study.

As shown in Figure 2, the talo-navicular coverage angle and calcaneal-first metatarsal angle taken from the initial normal-arched foot radiographs were used to calculate reference values for the flat-arched foot study. Participants qualified for the flat-arched study when both measures from the lateral and/or anterior-posterior views exceeded 1 SD from the actual mean values reported for the normal study. The decision to accept either the lateral or antero-posterior measurements was based on the lack of consensus regarding which plane best represents the 'flat-arched foot'.

### Reliability of clinical and radiological measures

The reliability of the clinical measurements has been reported to be moderate to excellent, with intra-class correlation coefficients (ICCs) of 0.67 and 0.99 for normalised navicular height [23] and the arch index [12], respectively. For radiographic measures, the ICCs are reported to be excellent for the calcaneal inclination angle (0.98), calcaneal-first metatarsal angle (0.99) [12] and good for the talo-navicular coverage angle (0.79) [24]. As the reliability of the talus-second metatarsal angle is unknown, we evaluated intra- and inter-tester reliability for this angle. Intra-tester reliability was evaluated by a podiatrist with seven years of post-graduate experience. Inter-tester reliability was evaluated between the same tester and one other tester with four years of undergraduate podiatry training. The x-ray measurements were marked onto clear-plastic overhead transparencies placed over the x-ray using a permanent fine-point marker. For intra-tester reliability, the tester was blinded from the initial measurements when they performed their re-test session approximately two-weeks later. For inter-tester reliability, the examiners evaluated the x-rays independently, were blinded to each other's assessment and the data for each angle was recorded from single measurements. Testers were not blinded from the participants' anthropometric measurements (e.g. clinical measures of foot posture) for either the intra-tester or intra-tester components of the study.

### Statistical analysis

To satisfy the assumption of independence with statistical analysis, only measurements from a single foot were analysed [25]. All data were explored for normal distribution by evaluating skewness and kurtosis. The relative reliability of the talo-navicular coverage angle was assessed using type (3,1) intra-class correlation coefficients and absolute limits of agreement [26]. To evaluate the anthropometric-related differences between the normal-arched and flat-arched groups, a series of independent-samples *t*-tests were used. To determine the degree of association between clinical and radiographic measurements, data from the normal-arched, flat-arched and non-qualifying groups were pooled and Pearson *r *correlation coefficients were calculated. For both the *t*-tests and correlation coefficients, the level of significance was set at 0.05. All statistical tests were conducted using SPSS version 13 for Windows (SPSS Inc, Chicago, IL).

## Results

### Participant characteristics

**Figure 6:**
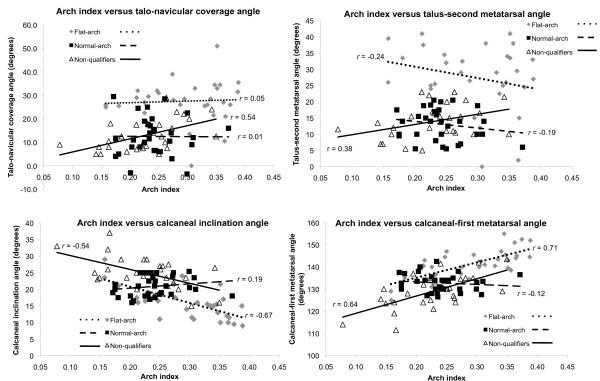
**Arch index versus radiographic measures for each foot posture group**. Scatter plots with trend lines for the arch index and radiographic measures of foot posture show the distribution of values for normal-arch, flat-arch and non-qualifying foot postures.

**Figure 7:**
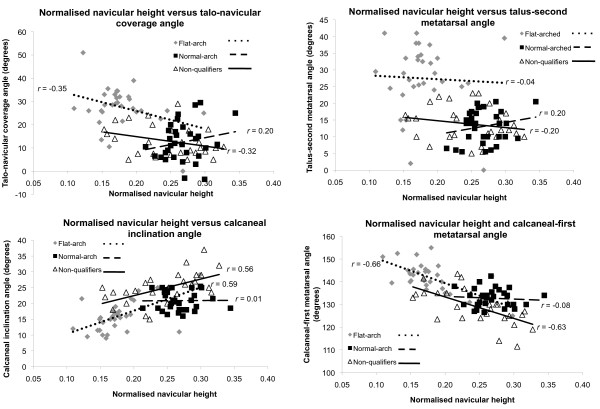
**Normalised navicular height versus radiographic measures for each foot posture group**. Scatter plots with trend lines for the normalised navicular height and radiographic measures of foot posture show the distribution of values for normal-arch, flat-arch and non-qualifying foot postures.

The mean ± SD age, height and body mass of the study sample were 23.2 ± 5.6 years, 1.70 ± 0.10 m, and 71.6 ± 14.6 kg, respectively. Following the radiographic assessment, 32 participants were recruited to the normal-arched study, 31 qualified for the flat-arched foot study and 28 participants were classified as having neither normal- or flat-arched feet and were not suitable for either study. Anthropometric data for the normal-arched, flat-arched and non-qualifying participants are summarised in Table 1. Scatter plots of the distributions of all participants' clinical and radiological measurements are shown in Figure 6 and 7.

### Reliability of the talus-second metatarsal angle

The within- and between-tester reliability of measuring the talus-second metatarsal angle is shown in Table 2. The talus-second metatarsal angle demonstrated good to excellent intra-rater reliability with left and right foot ICCs ranging from 0.71 to 0.91 and absolute random error ranging from 7.1 to 12.2°. Inter-rater reliability for the talus-second metatarsal angle was moderate to very good with left and right foot ICCs ranging from 0.68 to 0.78 and absolute random error ranging from 5.6 to 7.1° (Table 2).

**Table 2:** Relative and absolute reliability of measuring the talus-second metatarsal angle (T2MA)

	RELATIVE RELIABILITY	ABSOLUTE RELIABILITY	
		
	Type (3,1) ICC (95% CI)	Systematic bias (% mean difference)	Random error (95% LoA)
	
***Within-rater***			
left feet (n = 51)	0.91 (0.85 – 0.95)*	- 0.5°	7.1°
right feet (n = 51)	0.71 (0.55 – 0.83)*	- 0.3°	12.2°
			
***Between-rater***			
left feet (n = 41)	0.78 (0.62 – 0.88)*	- 1.0°	5.6°
right feet (n = 41)	0.68 (0.47 – 0.82)*	1.5°	7.1°

*Significant at *p *< 0.05

### Anthropometric differences between normal and flat-arched groups

General anthropometric characteristics including age, height and weight were not significantly different between the normal and flat-arched groups. However, all clinical and radiological differences were statistically different between groups (*p *< 0.001) (Table 1).

### Associations between clinical and radiological measures of foot posture

The relationships among the clinical and radiological measures (for the entire group n = 91) are shown in Table 3. Both clinical measures were significantly correlated with all radiographic angles, with *r *values ranging from 0.24 to 0.70. The clinical measurements displayed a moderate to strong relationship with radiographic measurement from the lateral view, with *r *values ranging from 0.59 to 0.70. However, the clinical measurements displayed only a weak to moderate relationship with radiographic measurement from the antero-posterior view, with *r *values ranging from 0.24 to 0.56. The strongest association between clinical and radiological measures occurred for the normalised navicular height and calcaneal first metatarsal inclination angle (*r *= 0.70). For the clinical measures, arch index and normalised navicular height displayed a significant negative correlation to each other (*r *= -0.58). For the radiographic measures, the lateral view angles were significantly correlated with angles obtained from the antero-posterior view, with *r *values ranging from 0.25 to 0.47. Figure 6 and 7 show scatter plots and associations between clinical and radiographic measures for each foot posture group.

**Table 3:** Pearson *r *values comparing the radiographic and clinical measures

	**Radiographic measures**				**Clinical measurements**	
		
	Lateral view		Anterior-posterior view			
	CIA	C1MA	TNCA	T2MA	AI	NNHt
		
**Clinical measurements**						
AI	- 0.59**	0.66**	0.40**	0.24*	-	- 0.58**
NNHt	0.60**	- 0.70**	- 0.56**	- 0.47**	-	-
						
**Radiographic measurements**						
Anterior-posterior view T2MA	- 0.25*	0.38**	-	-	-	-
TNCA	- 0.36**	0.47**	-	-	-	-

AI – arch index, NNHt – normalised navicular height truncated, CIA – calcaneal inclination angle, C1MA – calcaneal first metatarsal angle, TNCA – talo-navicular coverage angle, T2MA – talus-second metatarsal angle.

*Significant at *p *< 0.05, **Significant at *p *< 0.01

## Discussion

The purpose of developing this screening protocol was to assist with the recruitment of participants into a series of laboratory-based gait studies investigating functional differences between normal-arched and flat-arched feet. For the normal-arched study, the clinical and radiographic values were derived from two published sources [7,10], which describe normative foot posture in healthy and asymptomatic adult populations. Radiographic values obtained from the normal-arched foot study were subsequently used to calculate inclusion values for the flat-arched foot study. This resulted in normal and flat-arched groups with significantly different foot posture characteristics without systematic bias for age, height or weight between the groups.

**Figure 8:**
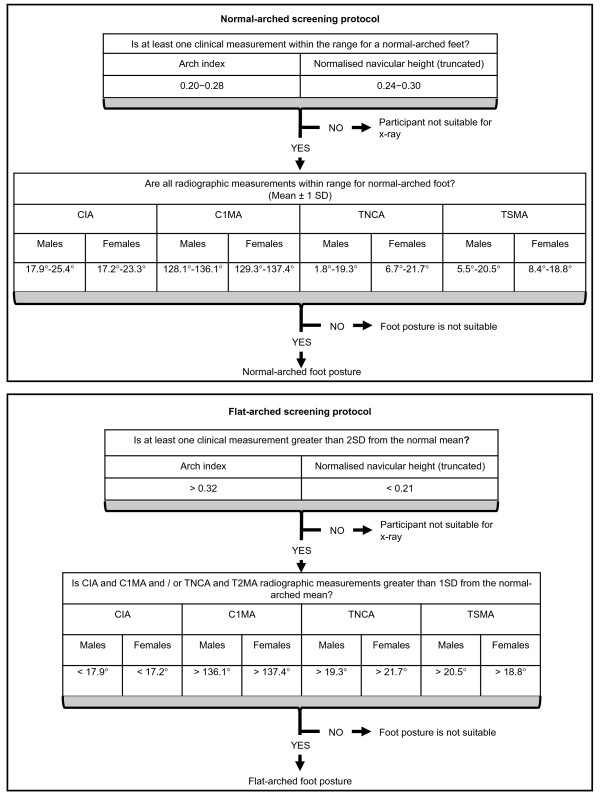
**Screening protocol for normal- and flat-arched foot posture**. Flow chart shows how the foot posture screening protocol can be applied to future studies recruiting participants with normal- and flat-arched foot posture. CIA – calcaneal inclination angle, C1MA – calcaneal-first metatarsal angle, TNCA – talo-navicular coverage angle, T2MA – talus-second metatarsal angle.

Participants with normal-arched feet in this study displayed a similar mean arch index value (0.24 ± 0.04) to those reported by Cavanagh and Rodgers [9] (0.23 ± 0.05) for 107 subjects (mean age, 30 years). Interestingly, our study found a higher mean arch index value (0.24 ± 0.04) compared to Scott and colleagues [7] (0.18 ± 0.07), from which our normative reference values were derived. This difference may be due to our study reporting arch index values from only participants who satisfied the radiographic inclusion criteria and not the full range of participants who underwent clinical screening. Accordingly, we recommend using the values from our study tabulated in Figure 8, as our normative arch index values were validated with radiographs.

It is difficult to compare the arch index values used to define the participants with flat-arched feet in our study (0.30 ± 0.07) to those of Cavanagh and Rodgers [9] (≥ 0.26), as they defined the 'flat-arched foot' to lie within the top 25% of the distribution of arch index scores obtained from the 107 subjects. In contrast, we defined the flat-arched foot as greater than two standard deviations from the normative mean (as reported by Scott and colleagues [7]). The rationale for using two standard deviations was to increase the likelihood of participants with flat-arched feet qualifying for inclusion via radiographic appraisal. Therefore, it is important to highlight that the arch index reference values that defined flat-arched feet in our study were stricter, which resulted in the recruitment of flatter-arched feet compared to those reported by Cavanagh and Rodgers [9].

From the normal-arched feet, we report the first normative values published for the calcaneal-first metatarsal angle and talo-navicular coverage angle from a young adult population (Table 1). The actual values obtained for the calcaneal inclination angle and talus-second metatarsal angle from normal-arched feet in this study were within 1.4° to 2.9°, respectively, of those reported by Thomas and colleagues [10] for 100 subjects (mean age, 35 years).

With respect to the relationship between clinical and radiographic measures, all correlations were statistically significant, with the associations ranging from moderate to strong (*r *= 0.24 to 0.70). Of the two clinical measures, normalised navicular height provided the strongest association with all radiographic angles measured from both the A-P and lateral views. These findings are different to the associations reported by Menz and Munteanu [12] who reported the arch index to provide the strongest correlation for the calcaneal inclination angle and calcaneal-first metatarsal angle from 95 older participants (mean age, 79 years). This discrepancy may be due to age-related differences in body mass of younger compared to older adult populations, as the arch index is confounded by variations in soft tissue composition of the foot between different individuals [27].

Furthermore, while both clinical measures were significantly correlated with all radiographic angles, the arch index and normalised navicular height were most strongly associated with the calcaneal inclination angle and calcaneal-first metatarsal angle obtained from the lateral view. Therefore, we found the arch index and normalised navicular height measurements were more sensitive to detecting flat-arched feet associated with angles measured from the lateral view, which better represents sagittal plane alignment. Consequently, using the arch index and normalised navicular height measurements in the current study may have lead to a bias when recruiting participants with flat arches characterised by a low calcaneal inclination angle and high calcaneal-first metatarsal angle. Further research is required to validate a reliable clinical test that is sensitive to radiographic variations with transverse plane deformity, such as the recently reported foot mobility magnitude test [28]. It is also not clear whether foot posture variations in the sagittal, transverse or both planes provide the best descriptor of the flat-arched foot. For example, loss of the tibialis posterior tendon function with disease is associated with abnormal joint moments in both the sagittal and transverse midfoot planes [29,30]. Ness et al. [29] reported significantly less forefoot plantarflexion and less abduction during walking in 34 patients with tibialis posterior tendon dysfunction compared to 25 healthy controls. This would indicate that an acquired flatfoot deformity is characterised by altered foot posture in multiple planes. However, the variants of foot posture investigated in our study present a different set of considerations because pain and dysfunction were not present.

The protocol for screening foot posture described here could be applied to future research studies specifically recruiting participants with normal- and flat-arched foot posture. With the moderate correlation between clinical and radiographic measures of foot posture, we recommend the arch index and normalised navicular height measurements be used during initial foot screening to identify potentially suitable participants, followed by radiographic evaluation including lateral and antero-posterior views.

This foot screening protocol needs to be viewed in light of some limitations. The intra- and inter-tester reliability of the talus-second metatarsal angle ranged from moderate to excellent with ICCs between 0.68 and 0.91 and limits of agreement ranging from 5.6° to 12.1°, respectively. Another drawback from this study is that the homogeneity of the participant group in this investigation limits the generalization of our findings to a young adult population.

Further research is required to provide validation of radiographic measures of foot posture by investigating whether the radiographic angles are related to functional differences during gait. Moreover, large prospective studies investigating the relationship between radiographic measures of foot posture and injury could provide further validation of the radiographic measures.

## Conclusion

The foot screening protocol presented here provides a strategy for recruiting participants with normal- and flat-arched foot posture, including reference values for clinical and radiographic measurement. The arch index and normalised navicular height ratios provide valid and reliable measures of foot posture. Normalised navicular height displayed the strongest association with radiographic angles, especially the calcaneal inclination angle. Further research is required to determine whether foot posture variations in the sagittal, transverse or both planes provide the best descriptor of the flat-arched foot. In the absence of this research, we recommend the protocol outlined in this article to classify foot posture in research.

## Competing interests

HBM and KBL are Editor-in-Chief and Deputy Editor-in-Chief, respectively, of *Journal of Foot and Ankle Research*. It is journal policy that editors are removed from the peer review and editorial decision-making processes for papers they have co-authored.

## Authors' contributions

GSM, HBM and KBL conceived the idea and obtained funding for the study. GSM, HBM and KBL designed the study protocol. GSM recruited/screened participants' foot posture and evaluated the radiographs. GSM, HBM and KBL drafted the manuscript. All authors have read and approved the final manuscript.

## References

[B1] Redmond AC Crane YZ Menz HB Normative values for the Foot Posture Index J Foot Ankle Res 2008 2 6 10.1186/1757-1146-1-6 18822155 PMC2553778

[B2] Redmond AC Crosbie J Ouvrier RA Development and validation of a novel rating system for scoring standing foot posture: The Foot Posture Index Clin Biomech (Bristol, Avon). 2006 2 1 89 98 10.1016/j.clinbiomech.2005.08.002 16182419

[B3] Hunt AE Smith RM Mechanics and control of the flat versus normal foot during the stance phase of walking Biomech (Bristol, Avon) 2004 2 4 391 397 10.1016/j.clinbiomech.2003.12.010 15109760

[B4] Murley GS Landorf KB Menz HB Bird AR Effect of foot posture, foot orthoses and footwear on lower limb muscle activity during walking and running: a systematic review Gait Posture 2009 2 172 187 10.1016/j.gaitpost.2008.08.015 18922696

[B5] Brewerton DA Sandifer PH Sweetnam DR "Idiopathic" Pes Cavus: an Investigation into Its Aetiology Br Med J 1963 2 659 661 10.1136/bmj.2.5358.659 14044868 PMC1872745

[B6] Keenan MA Peabody TD Gronley JK Perry J Valgus deformities of the feet and characteristics of gait in patients who have rheumatoid arthritis J Bone Joint Surg Am 1991 2 237 247 1993719

[B7] Scott G Menz H Newcombe L Age-related differences in foot structure and function Gait Posture 2007 2 68 75 10.1016/j.gaitpost.2006.07.009 16945538

[B8] McPoil TG Cornwall MW Vicenzino B Teyhen DS Molloy JM Christie DS Collins N Effect of using truncated versus total foot length to calculate the arch height ratio Foot 2008 2 220 227 10.1016/j.foot.2008.06.002 20307441

[B9] Cavanagh PR Rodgers MM The arch index: a useful measure from footprints J Biomech 1987 2 547 551 10.1016/0021-9290(87)90255-7 3611129

[B10] Thomas JL Kunkel MW Lopez R Sparks D Radiographic values of the adult foot in a standardized population J Foot Ankle Surg 2006 2 3 12 10.1053/j.jfas.2005.10.014 16399552

[B11] Menz HB Alternative techniques for the clinical assessment of foot pronation J Am Podiatr Med Assoc 1998 2 119 129 9542353 10.7547/87507315-88-3-119

[B12] Menz HB Munteanu SE Validity of 3 clinical techniques for the measurement of static foot posture in older people J Orthop Sports Phys Ther 2006 2 179 10.2519/jospt.2006.0201 16596894

[B13] Scharfbillig R Evans AM Copper AW Williams M Scutter S Iasiello H Redmond A Criterion validation of four criteria of the foot posture index J Am Podiatr Med Assoc 2004 2 31 38 14729988 10.7547/87507315-94-1-31

[B14] Saltzman CL Nawoczenski DA Talbot KD Measurement of the medial longitudinal arch Arch Phys Med Rehabil 1995 2 45 49 10.1016/S0003-9993(95)80041-7 7811174

[B15] Exposure of Humans to Ionizing Radiation for Research Purposes (2005) http://www.arpansa.gov.au/pubs/rps/rps8.pdf

[B16] Burns J Crosbie J Hunt A Ouvrier R The effect of pes cavus on foot pain and plantar pressure Clin Biomech (Bristol, Avon) 2005 2 877 882 10.1016/j.clinbiomech.2005.03.006 15882916

[B17] Burns J Keenan A-M Redmond A Foot Type and Overuse Injury in Triathletes J Am Podiatr Med Assoc 2005 2 235 241 15901809 10.7547/0950235

[B18] Cowan DN Jones BH Robinson JR Foot morphologic characteristics and risk of exercise-related injury Arch Fam Med 1993 2 773 777 10.1001/archfami.2.7.773 7906597

[B19] McCrory JL Young MJ Boulton AJM Cavanagh PR Arch index as a predictor of arch height Foot 1997 2 79 81 10.1016/S0958-2592(97)90052-3

[B20] Menz HB Morris ME Clinical determinants of plantar forces and pressures during walking in older people Gait Posture 2006 2 229 236 10.1016/j.gaitpost.2005.09.002 16214340

[B21] Beaglehole R Bonita R Kjellstrom T Basic epidemiology 1993 Geneva: World Health Organization

[B22] Sangeorzan BJ Mosca V Hansen ST Jr Effect of calcaneal lengthening on relationships among the hindfoot, midfoot, and forefoot Foot Ankle 1993 2 136 141 8491427 10.1177/107110079301400305

[B23] Menz HB Tiedemann A Kwan MM Latt MD Sherrington C Lord SR Reliability of clinical tests of foot and ankle characteristics in older people J Am Podiatr Med Assoc 2003 2 380 387 13130085 10.7547/87507315-93-5-380

[B24] Schon LC Weinfeld SB Horton GA Resch S Radiographic and clinical classification of acquired midtarsus deformities Foot Ankle Int 1998 2 394 404 9677084 10.1177/107110079801900610

[B25] Menz HB Two feet, or one person? Problems associated with statistical analysis of paired data in foot and ankle medicine Foot 2004 2 2 5 10.1016/S0958-2592(03)00047-6

[B26] Atkinson G Nevill AM Statistical methods for assessing measurement error (reliability) in variables relevant to sports medicine Sports Med 1998 2 217 238 10.2165/00007256-199826040-00002 9820922

[B27] Wearing SC Hills AP Byrne NM Hennig EM McDonald M The arch index: a measure of flat or fat feet? Foot Ankle Int 2004 2 575 581 15363380 10.1177/107110070402500811

[B28] McPoil TG Vicenzino B Cornwall MW Collins N Warren M Reliability and normative values for the foot mobility magnitude: a composite measure of vertical and medial-lateral mobility of the midfoot J Foot Ankle Res 2009 2 6 10.1186/1757-1146-2-6 19267907 PMC2656480

[B29] Ness ME Long J Marks R Harris G Foot and ankle kinematics in patients with posterior tibial tendon dysfunction Gait Posture 2008 2 331 339 10.1016/j.gaitpost.2007.04.014 17583511

[B30] Ringleb SI Kavros SJ Kotajarvi BR Hansen DK Kitaoka HB Kaufman KR Changes in gait associated with acute stage II posterior tibial tendon dysfunction Gait Posture 2007 2 555 564 10.1016/j.gaitpost.2006.06.008 16876415

